# Beta amyloid deposition and cognitive decline in Parkinson’s disease: a study of the PPMI cohort

**DOI:** 10.1186/s13041-022-00964-1

**Published:** 2022-09-13

**Authors:** Alexander S. Mihaescu, Mikaeel Valli, Carme Uribe, Maria Diez-Cirarda, Mario Masellis, Ariel Graff-Guerrero, Antonio P. Strafella

**Affiliations:** 1 Brain Health Imaging Centre, Campbell Family Mental Health Research Institute, Centre for Addiction and Mental Health, Toronto, ON Canada; 2 Krembil Brain Institute, University Health Network, University of Toronto, Toronto, ON Canada; 3 Institute of Medical Science, University of Toronto, Toronto, ON Canada; 4 Neurodegenerative Diseases Group, Biocruces Bizkaia Health Research Institute, Barakaldo, Spain; 5 LC Campbell Cognitive Neurology Research Unit, Sunnybrook Research Institute, University of Toronto, Toronto, ON Canada; 6 Hurvitz Brain Sciences Program, Sunnybrook Research Institute, Toronto, ON Canada; 7 Morton and Gloria Shulman Movement Disorder Unit & Edmond J. Safra Program in Parkinson Disease, Neurology Division, Department of Medicine, Toronto Western Hospital, University Health Network, University of Toronto, Toronto, ON Canada

**Keywords:** Parkinson’s disease, Beta amyloid, Cognitive decline, [18F]Florbetaben, PPMI

## Abstract

The accumulation of beta amyloid in the brain has a complex and poorly understood impact on the progression of Parkinson’s disease pathology and much controversy remains regarding its role, specifically in cognitive decline symptoms. Some studies have found increased beta amyloid burden is associated with worsening cognitive impairment in Parkinson’s disease, especially in cases where dementia occurs, while other studies failed to replicate this finding. To better understand this relationship, we examined a cohort of 25 idiopathic Parkinson’s disease patients and 30 healthy controls from the Parkinson’s Progression Marker Initiative database. These participants underwent [^18^F]Florbetaben positron emission tomography scans to quantify beta amyloid deposition in 20 cortical regions. We then analyzed this beta amyloid data alongside the longitudinal Montreal Cognitive Assessment scores across 3 years to see how participant’s baseline beta amyloid levels affected their cognitive scores prospectively. The first analysis we performed with these data was a hierarchical cluster analysis to help identify brain regions that shared similarity. We found that beta amyloid clusters differently in Parkinson’s disease patients compared to healthy controls. In the Parkinson’s disease group, increased beta amyloid burden in cluster 2 was associated with worse cognitive ability, compared to deposition in clusters 1 or 3. We also performed a stepwise linear regression where we found an adjusted R^2^ of 0.495 (49.5%) in a model explaining the Parkinson’s disease group’s Montreal Cognitive Assessment score 1-year post-scan, encompassing the left gyrus rectus, the left anterior cingulate cortex, and the right parietal cortex. Taken together, these results suggest regional beta amyloid deposition alone has a moderate effect on predicting future cognitive decline in Parkinson’s disease patients. The patchwork effect of beta amyloid deposition on cognitive ability may be part of what separates cognitive impairment from cognitive sparing in Parkinson’s disease. Thus, we suggest it would be more useful to measure beta amyloid burden in specific brain regions rather than using a whole-brain global beta amyloid composite score and use this information as a tool for determining which Parkinson’s disease patients are most at risk for future cognitive decline.

**Supplementary Information:**

The online version contains supplementary material available at 10.1186/s13041-022-00964-1.

## Introduction

Parkinson’s disease (PD) is the second most common neurodegenerative disorder, presenting with progressively deteriorating motor and cognitive symptoms [1, 2]. While the etiology of the motor deficits is generally understood [3, 4], the underlying cause of cognitive decline in PD remains elusive [1, 5]. Numerous mechanisms of action have been proposed such as neurotransmitter system dysregulation, neuroinflammation and abnormal deposition of proteinopathies [6, 7, 8, 9]. Among the latter is the neurotoxic deposition in the brain of beta amyloid (Aβ)  [10] which was previously found to contribute specifically towards the cognitive dysfunction symptoms in PD [11]. Despite the wealth of research, the role of Aβ in PD cognitive decline remains controversial with some studies finding no difference in Aβ burden when comparing PD patients with and without cognitive decline [12, 13].

Cognitive decline in PD is a distressing sequela of the disease associated with poorer quality of life and increased caretaker burden [14]. PD cognitive decline typically progresses over the disease course, with 21–24% of newly diagnosed PD patients having mild cognitive impairment (PD-MCI) at time of diagnosis [15] and upwards of 80% of PD patients eventually developing PD dementia (PDD) within 20 years of PD diagnosis [16]. Notably, however, other PD patients remain cognitively unimpaired (PD-CU) for years [17, 18, 19]. Understanding why some PD patients progress to PDD and others remain cognitively stable long-term is an important first step in finding future targets for symptom intervention.

The hypothesis that Aβ has a role in PD cognitive decline has been fueled by studies which found elevated levels of Aβ deposits in the brains of PDD patients at levels that sometimes resemble advanced Alzheimer’s disease dementia in both symptom and protein profiles [20, 21]. Siderowf and colleagues [22] found that lower levels of Aβ in the cerebrospinal fluid (CSF) of PD patients was associated with increased future cognitive decline in PD patients, as measured by a dementia rating scale at follow-ups. Studies have established that lower Aβ in the CSF is strongly correlated with increased cortical Aβ burden; in the brain, Aβ forms extracellular neurotoxic fibrils which are less able to diffuse back into the CSF [23, 24]. Elevated Aβ in the brain is also considered to be a normal part of aging, with 20–35% of otherwise healthy seniors having elevated Aβ burden in their brains [25]. Conversely, inhibiting endogenous Aβ production in ex vivo neuronal tissue samples kills the neurons [26, 27] so a ‘Goldilocks’ moderate amount of Aβ is likely needed for healthy brain function [28].

Although previous studies have examined the role of Aβ in PD cognitive decline, these studies have largely attribute amyloidpathy as a whole-brain binary outcome with the entire brain being either above or below a diagnostic cut-off value for Aβ levels: either “Aβ positive” (Aβ+) or “Aβ negative” (Aβ−) [13, 29]. Instead, our study will examine the role Aβ burden has on cognitive performance using a scalar Aβ measurement and examining local features of Aβ deposition using cortical regions of interest (ROIs). Akhtar and colleagues [30] were one of the first investigators to suggest that regional Aβ binding in the brain is more important for identifying cognitive outcomes in PD than global Aβ values. Their study found amyloid positivity was not associated with PD-MCI status, however, increased Aβ binding in different brain regions correlated with specific neurological test scores: [^18^F]Florbetapir standard uptake value ratio (SUVR) in the posterior cingulate cortex (PCC) was inversely correlated with verbal memory performance while SUVRs in the frontal cortex, precuneus, and anterior cingulate cortex (ACC) were inversely correlated with naming performance. Where Aβ is deposited is as important as how much Aβ is deposited.

Our study measured in vivo Aβ deposition in the brain using positron emission tomography (PET) with the radiotracer [^18^F]Florbetaben ([^18^F]FBB), which can measure Aβ density in cortical brain regions [31]. We used imaging and neuropsychological test data from the open-source database Parkinson’s Progression Marker Initiative (PPMI) to see how Aβ burden in different brain areas impacts cognitive function using a cohort of longitudinally assessed PD participants. We hypothesized that Aβ burden would have a negative correlation with cognitive function in PD, but only when examining specific cortical ROI, and not at the global level with Aβ categorized as positive or negative. Rather than suggest cognitive decline in PD is entirely Aβ driven or not, our study aimed to describe cognitive decline can be attributed to Aβ deposition, and which brain areas are most susceptible.

## Materials and methods

### Participants

Data used in the preparation of this article were obtained from the Parkinson’s Progression Markers Initiative (PPMI) database (www.ppmi-info.org/data). For up-to-date information on the study, visit www.ppmi-info.org. Enrollment into the PPMI dataset began on June 1st, 2010 and enrollment for the PD group was completed April 2013, with longitudinal data collection continuing at the time this article being written. All the participating PPMI sites received approval from an ethical standards committee on human experimentation before study commencement, received informed written consent from all participants in the study, and was in full compliance with the principles set out by the Declaration of Helsinki.

A total of 70 PD patients and 40 healthy controls (HC) were initially found to have [^18^F]FBB imaging data available as of March 31st, 2021. These subjects met the inclusion and exclusion criteria identified by the PPMI protocol which is available online on https://www.ppmi-info.org/sites/default/files/docs/PA2_PPMI_Clinical%20Protocol_Final_01Feb2021.pdf. For PD patients, inclusion criteria are listed online at the website provided in section 7.2.1 and exclusion criteria are listed in 7.2.2. For HC, inclusion criteria are listed in 7.1.1 and exclusion criteria are listed in 7.1.2

In addition to the inclusion and exclusion criteria set by the PPMI protocol, we further excluded subjects if they did not meet criteria specific to our study. Inclusion criteria specific for our study were: (1) both PD and HC must have full [^18^F]FBB imaging data for all 20 cortical ROIs, (2) age 50 years or older at time of [^18^F]FBB scan and, (3) participants must have a Montreal Cognitive Assessment (MoCA) score available at time of the [^18^F]FBB scan and at least one score in the 2-year follow-up period after scan. Exclusion criteria included: (1) PD patients which had a β-glucocerebrosidase (GBA), α-Synuclein **(**SNCA), or leucine-rich repeat kinase 2 (LRRK2) variant allele with a known link to PD predisposition (see Additional file 1: Table S1 for a list of the alleles excluded), (2) PD patients with an unknown apolipoprotein E (APOE) status due to this protein’s strong association with Aβ deposition [11, 32], and (3) HC who did not have a MoCA score of 26 or higher at the time of [^18^F]FBB scan.

After applying these inclusion and exclusion criteria, we had a final sample of 25 idiopathic PD patients and 30 HCs at the time of [^18^F]FBB scan. The PD patients were also confirmed not pathogenic in the three genes (i.e., GBA, SNCA, and SLRRK2) by a PPMI consensus committee.

### Clinical assessments and neuropsychological testing

Demographic information we assessed included sex, age at scan, and years of education. Clinical assessments included the Movement Disorders Society‐Unified Parkinson Disease Rating Scale (MDS‐UPDRS) part III motor section [33] both “ON” and “OFF” medication, Hoehn and Yahr (H&Y) score [34], the Geriatric Depression Scale (GDS) [35], disease duration, and levodopa equivalent daily dose (LEDD). Global cognition was assessed with the MoCA [36]. The MoCA is a widely available and quick to administer test with good sensitivity for detecting cognitive impairment in PD [37]. Clinical and cognitive assessments were conducted at time of [^18^F]FBB scan and additional cognitive assessments were performed yearly for 2 years after scan.

We used a MoCA score ≤ 25 as cut-off point for MCI classification for PD and HC which has shown good sensitivity and specificity [38, 39]. All HC had a MoCA score of 26 or higher at time of [^18^F]FBB scan. Using this MoCA cut-off of ≤ 25 as a classification for PD-MCI, 7 out of a total of 25 (28%) PD patients were PD-MCI at time of scan, 5 out of 23 (21.7%) PD patients were PD-MCI 1 year after scan, and 4 out of 21 (19%) PD patients were PD-MCI 2 years after scan. The fewer amount of PD patients 1- and 2-years after [^18^F]FBB scan is due to some PD patients dropping out of the PPMI study at their follow-up appointments. In the HC group, no participants were MCI at time of scan as per our inclusion criteria, 2 out of a total of 18 (11.1%) HCs were MCI 1 year after scan and 1 out of 17 (5.9%) HCs was MCI 2 years after scan. Some HCs did not return for their 1-year follow-up but did return for their 2-year follow-up, while other HCs dropped out after their first follow-up.

### [^18^F]FBB image acquisition and pre-processing

[^18^F]FBB PET images were acquired at approved PPMI centers in accordance with a standardized [^18^F]FBB imaging protocol (see https://www.ppmi-info.org/wp-content/uploads/2017/07/PPMI_FBB-PET-TOM_V3_09-March-2017.pdf). The [^18^F]FBB scans were performed using either a GE or a SIEMENS PET scanner (GE-DLS, GE Discovery 710, Siemens Biograph 6, Siemens HR+). Images were scanned in a 128 × 128 matrix size and post reconstruction filter of a Gaussian FWHM 5.0 mm was applied. Participants received the [^18^F]FBB injection as a single intravenous bolus injection consisting of 300 MBq (± 20%) in the antecubital region, followed by a flush of 0.9% sodium chloride to ensure the full radiotracer dose is administered to each participant. Participants rested for 80 min, a 10-min attenuation correction was performed, and then a 4 × 5-min emission scan was obtained. Participants were at rest and had their heads secured by Velcro during the PET scan.

[^18^F]FBB PET images were first assessed for quality control at an imaging lab (Institute for Neurodegenerative Disorders, New Haven, Connecticut) and then imported to PMOD Biomedical Image Software (PMOD Technologies, Zurich, Switzerland) for PET image processing. Motion correction was first applied to the dynamic PET frames if needed and then an average time-weighted PET frame was created. This averaged frame was normalized to standard Montreal Neurological Institute (MNI) space and then converted to standard uptake values (SUVs). Volumes of interest (VOIs) from the Automated Anatomical Labeling (AAL) single-subject atlas were merged and applied to the SUV volumes of each participant, adjusted for individual brain atrophy [40]. Semi-quantitative measurements in the form of average SUV per voxel were extracted from the saved individual VOIs and used to create regional SUV ratios (SUVR). The mean cerebellar grey matter cortex was used as the reference region for this ratio. The SUVR values for the 20 bilateral cortical ROIs were downloaded from the PPMI database and used in the present study (see Table 1 for a list of the ROIs included).

**Table 1:** List of the 20 bilateral cortical regions of interest included in the study

List of regions of interest	
Left frontal cortex	Right frontal cortex
Left orbitofrontal cortex	Right orbitofrontal cortex
Left gyrus rectus	Right gyrus rectus
Left anterior cingulum	Right anterior cingulum
Left posterior cingulum	Right posterior cingulum
Left mesial temporal cortex	Right mesial temporal cortex
Left temporal cortex	Right temporal cortex
Left lateral temporal cortex	Right lateral temporal cortex
Left parietal cortex	Right parietal cortex
Left occipital cortex	Right occipital cortex

### Statistical analyses

Statistical significance of any group differences between the PD and HC groups regarding their demographic measurements was calculated using SPSS software (SPSS Statistics 27; IBM Corp. Armonk, NY, USA). A two-sample t test (*p* < 0.05) was used to compare PD and HC groups for the continuous variables of age, years of education, MoCA and GDS. A chi-square (*p* < 0.05) was used to compare the sex ratio between the two groups.

### Hierarchical cluster analysis

**Fig. 1:**
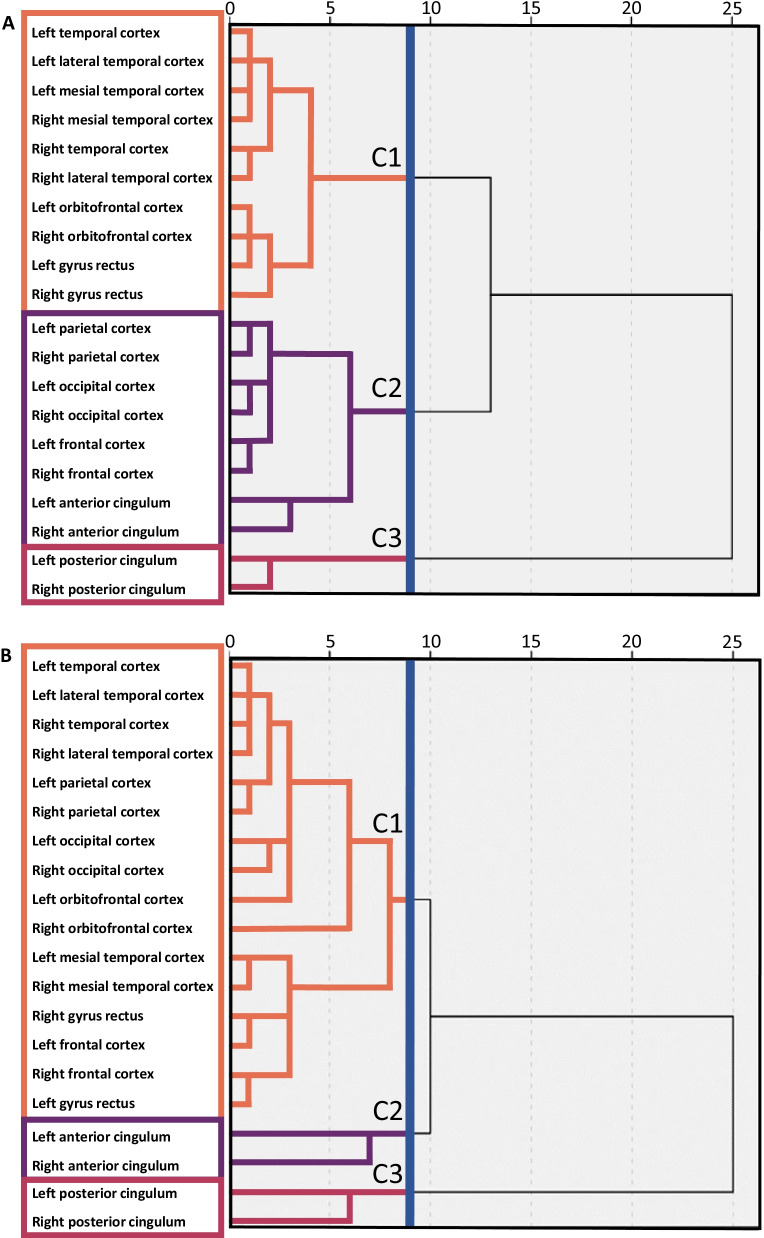
Dendrograms of the clustering solutions for the [^18^F]Florbetaben standardized uptake value ratios of each of the 20 regions of interest for the **A** Parkinson’s disease patient group on the left and the **B** healthy control group on the right. The blue line shows the cut-off solution with three clusters. The three clusters are colour coded in orange for cluster 1 (C1), purple for cluster 2 (C2), and red for cluster 3 (C3)

Agglomerative hierarchical cluster analysis was performed using SPSS software (SPSS Statistics 27; IBM Corp. Armonk, NY, USA). We used the [^18^F]FBB SUVR values of the 20 ROIs in the PD and HC groups respectively to form two group dendrograms in an unsupervised manner (Fig. 1). This method analyzed the data in a ‘bottom-up’ manner: we used Ward’s clustering linkage method [41] which begins with all 20 ROIs as their own cluster. The algorithm then combines clusters stepwise to minimize the variance within the clusters, measured by the sum of squares index. The algorithm is based on the premise that merging two clusters will decrease the similarity of cluster members and thus tries to minimize this dissimilarity i.e., the merging cost. At each step, every possible combination of clusters is tested before new larger clusters are established which have the minimum increase of within cluster variance. These new larger clusters are then iteratively tested and combined again to minimize the variance once more, repeating this process of joining clusters together until only one cluster containing all the ROIs remains. Ward’s method is a popular clustering algorithm that maximizes the differences between clusters while also maximizing the similarity within clusters. Moreover, the hierarchical clustering method allows us to select a different number of clusters as our solution simply by selecting a different cut-off point in the dendrogram [42, 43, 44].

We calculated the Calinski-Harabasz index with MATLAB to select the number of clusters we should use as the cut-off solution. Each cluster cut-off solution has a Calinski-Harabasz value which looks to maximize between-cluster variance while minimizing within-cluster variance. The larger the Calinski-Harabasz ratio, the better the solution [44] (https://es.mathworks.com/help/stats/clustering.evaluation.calinskiharabaszevaluation-class.html).

A Pearson correlation coefficient was calculated for the SUVRs in the 20 ROIs at each of the three different years and the corresponding MoCA scores of that year (‘SUVR-ROIs’ × ‘MoCA scores’) in both the PD and HC groups. These coefficients were grouped together by the clusters found in the previous analysis and were not corrected for multiple comparisons.

### Stepwise linear regression modeling

We modelled a stepwise linear regression analysis in SPSS (SPSS Statistics 27; IBM Corp. Armonk, NY, USA) using the MoCA scores at each of the three different time points as the dependent variable and [^18^F]FBB SUVR values in 20 cortical ROIs at year of scan as the independent variables for both PD and HC separately. This method uses ‘top-down’ analysis, beginning with all 20 ROIs as potential variables in the model. The algorithm then does multiple regression analyses at once, each time removing the weakest correlated independent variable, in this case an ROI. In the end, only the ROIs that explain MoCA score best are included in a model. The model we presented for each group is the one that maximizes the adjusted R^2^ value for each year of MoCA score, which means the model that explains the greatest amount of variance in the MoCA score. The adjusted R^2^ shrinks the R^2^ with consideration of the number of predictors and sample size in the model [45]. The adjusted R^2^ can be converted into a percentage value which explains that percentage of the variance. For example, an adjusted R^2^ of 0.5 would explain 50% of the variance in that model.

The independent variables should be normally distributed, and no relationship should exist between the independent variables (known as collinearity). Normality was tested with a Shapiro-Wilks test using SPSS (SPSS Statistics 27; IBM Corp. Armonk, NY, USA) for each ROI found in a regression model. Collinearity was tested with a Durbin-Watson test of collinearity using SPSS (SPSS Statistics 27; IBM Corp. Armonk, NY, USA) (see Additional file 2: Results, for more details regarding these tests).

We also modelled the data using the APOE status of the PD patients as an additional covariate using two methods. Most participants in the HC group did not have their APOE status measured and thus could not be analyzed. The first method included the APOE status of each PD patient as a separate dependent variable which can be dropped from the model like the other 20 ROIs if it is not significant to the model. The second method we used added the APOE status as an additional dependent variable to any model we found with the stepwise method. The APOE status and any ROIs would then be modelled using an ‘enter’ method which does not drop any variables to see how the addition of the APOE status would affect the model. The APOE status was codified as a value ranging from 4 to 8. Every participant has a pair of APOE alleles which can each be either E2, E3 or E4. We coded a E2 as 2, E3 as 3, and E4 as a 4; then the two allele were summed together for each participant for their APOE value i.e., an E3/E4 would be coded as a 7.

## Results

### Demographics and clinical characteristics

Detailed demographic and clinical information of the 25 PD participants and 30 HCs at time of scan is shown in Table 2. No data were missing for age, sex, GDS, and years of education for either group and no data were missing for disease duration, MDS-UPDRS-III, and H&Y score for the PD group. Using a two-sample t-test comparing the PD group to HC, the only difference between PD and HC was found in MoCA score, t(36.114) = − 2.102; n = 55; *p* = 0.043. We did not find any group differences in terms of age, t(40.346) = 0.927; n = 55, *p* = 0.36, years of education, t(53) = − 1.400; n = 55; *p* = 0.167, or GDS, t(53) = 0.835; n = 55; p = 0.407. A *chi*-square test for the nominal variable of sex showed a significant relationship between the two groups in terms of sex ratio, *X*^2^ (1, N = 55) = 8.213, *p* = 0.004; however, there is insufficient females in the PD group (N_female_ = 4) to further explore this relationship.

**Table 2:** Participant demographics and clinical characteristics for the Parkinson’s disease and healthy control groups at time of [^18^F]Florbetaben scan

	Parkinson’s disease	Healthy controls
Population (female)	25 (4 female)	30 (17 female)^a^
Age (years)	67.56 [8.74]	65.67 [5.80]
Education (years)	16.20 [2.90]	17.40 [3.37]
MoCA	27.04 [2.46]	28.20 [1.37]^b^
GDS	5.06 [1.03]	4.93 [0.94]
Disease duration (months)	50.04 [16.91]	n/a
UPDRS-III ON	25.70 [8.94]	n/a
UPDRS-III OFF	29.92 [8.89]	n/a
LEDD (mg)	345.19 [217.39]	n/a

Values are expressed as a mean where appropriate with the standard deviation in square brackets

^a^Difference found between PD and HC at *p* < 0.05 using a chi-square test

^b^Difference found between PD and HC at *p* < 0.05 using an independent samples *t* test

The APOE status was available for all 25 PD patients, with one PD patient who was E3/E2, 17 were E3/E3, six were E4/E3, and one was E4/E4. In the HC group, nine out of the total 30 had their APOE status confirmed, of which three were E2/E3 and six were E3/E3, with no E4 alleles in the HC group.

### Hierarchical cluster analysis

We performed a cluster analysis to help identify which brain regions shared similarity in their Aβ deposition. A dendrogram for the hierarchical cluster analysis using Ward’s linkage method for PD and HC are shown in Fig. 1A, B, respectively. A three-cluster solution was chosen for both PD and HC which had the second-highest Calinksi-Harabasz values. The highest value was the two-cluster solution which led to the bilateral PCC being in its own cluster and the other 18 ROIs in a second cluster for both PD and HC groups. Thus, we selected the three-cluster solution for better discriminative utility between ROIs.

**Fig. 2:**
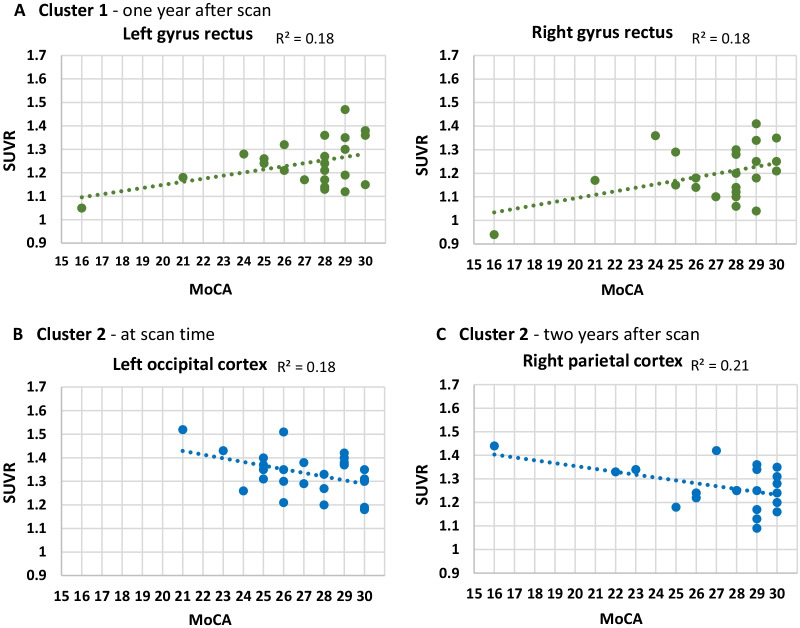
Parkinson’s disease patients: Cluster 1 and 2 scatterplots showing the [^18^F]Florbetaben ‘standardized uptake value ratios (SUVR)’ × ‘Montreal Cognitive Assessment scores (MoCA)’ for the regions of interest that have statistically significant Pearson correlations. Scatterplots are coloured blue for a negative relationship and green for a positive relationship between the two variables

The ROIs in PD patients correlated more strongly with neighbouring brain regions (Fig. 1A) and two of the clusters had at least 8 ROIs in each. Cluster 1 in PD consisted of the temporal lobe regions and the bilateral orbitofrontal cortex and bilateral gyrus rectus from the frontal region (Fig. 1A). The Pearson correlation analysis of the cluster 1 (SUVR-ROIs × MoCA scores) had a mixed effect (see Additional file 3: Fig. S1A for the Pearson correlation values in the PD group). Within cluster 1, the bilateral gyrus rectus had a statistically significant positive correlation with MoCA score 1 year after scan (Fig. 2A), wherein a higher [^18^F]FBB SUVR in these regions correlated with a higher MoCA score. This implied that, for these frontal regions, cognitive performance was not influenced by Aβ levels. The significance for the Pearson correlations was set to *p* < 0.05 uncorrected as an exploratory investigation of the cluster groupings and none of the ROIs survived correction for multiple comparisons.

Cluster 2 in PD consisted of the bilateral parietal cortex, bilateral frontal cortex, bilateral occipital cortex, and bilateral anterior cingulate cortex (Fig. 1A). Within cluster 2, the left occipital cortex at time of scan (Fig. 2B) and the right parietal cortex 2 years after scan (Fig. 2C) had a significant negative correlation with MoCA score, wherein a higher [^18^F]FBB SUVR in these brain regions was associated with poorer cognitive performance.

**Fig. 3:**
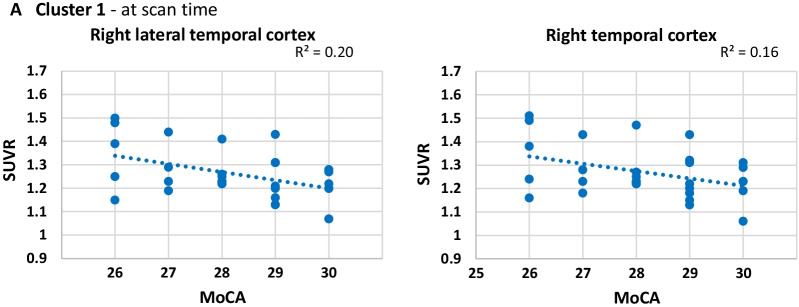
Healthy controls: Cluster 1 scatterplots showing the [^18^F]Florbetaben ‘standardized uptake value ratios (SUVR)’ × ‘Montreal Cognitive Assessment scores (MoCA)’ for regions of interest that have statistically significant Pearson correlations at baseline time of scan. Scatterplots are coloured blue for a negative relationship

In HC, all ROIs but the bilateral ACC and PCC correlated very strongly with each other, forming a large cluster, cluster 1, with 16 ROIs (Fig. 1B). Cluster 2 and cluster 3 were small, with only 2 ROIs in each, consisting of the bilateral ACC and bilateral PCC, respectively. The Pearson correlation analysis of the three clusters (SUVR-ROIs × MoCA scores) are shown in Additional file 3: Fig. S1B, for the HC group. The HC group was not normally distributed so the Spearman correlation was also calculated for this group in Additional file 3: Fig. S1C. Within cluster 1, the right temporal cortex and right lateral temporal cortex had a significant negative Pearson correlation with MoCA score at time of scan (Fig. 3A), implying that lower [^18^F]FBB SUVR in these brain regions was associated with better cognitive performance. The right lateral temporal cortex ROI was also found in the Spearman correlation.

In both the PD and HC groups, the bilateral PCC formed a cluster on its own (cluster 3) (Fig. 1A, B).

### Linear regression

We modelled a stepwise linear regression analysis to investigate which ROI(s) would best explain MoCA scores for each of the 3 years.

In PD (Table 3), a different model was found for each of the 3 years. At time of scan, the stepwise linear regression found a model consisting of the left occipital cortex with an adjusted R^2^ of 0.148 (14.8%) and a p-value of 0.033. One year after scan, the stepwise linear regression found a model consisting of the left gyrus rectus, right parietal cortex and left anterior cingulate cortex with an adjusted R^2^ of 0.495 (49.5%) and a p-value of 0.001. Two years after scan, the stepwise linear regression found a model consisting of the left gyrus rectus and right parietal cortex with an adjusted R^2^ of 0.319 (31.9%) and a p-value of 0.012. All ROIs in the PD models were normally distributed and passed the Durbin-Watson test of collinearity (see Additional file 2: Results, for more information on the Durbin-Watson test). Overall, the stepwise linear regression analysis found models which explained between 14.8 and 49.5% of the variance in PD MoCA score across the 3 different years. Refer to Additional file 4: Table S2, for a table which shows the coefficient values of each ROI found in the PD group’s models.

**Table 3:** Stepwise linear regression showing the model with the highest adjusted R^2^ value for the PD group

Regions of interest	R^2^	Adjusted R^2^	Standard error of the estimate	*p*-value
MoCA at scan				
Left occipital cortex	0.183	0.148	2.269	0.033
MoCA 1 year after scan				
Left gyrus rectus, right parietal cortex, left anterior cingulum	0.564	0.495	2.298	0.001
MoCA 2 years after scan				
Right parietal cortex, right gyrus rectus	0.387	0.319	2.889	0.012

In HC (Table 4), we only found a linear regression model at time of the scan and no model was possible for 1 year or 2 years post-scan. At time of scan, the stepwise linear regression found a model consisting of the right lateral temporal cortex, right mesial temporal cortex, and right parietal cortex with an adjusted R^2^ of 0.337 (33.7%) and a p-value of 0.003. None of these ROIs were normally distributed however and caution should be used when interpreting these results. These ROIs did however pass the Durbin–Watson test of collinearity. Refer to Additional file 5: Table S3, for a table which shows the coefficient values of each ROI found in the HC group’s model.

**Table 4:** Stepwise linear regression showing the model with the highest adjusted R^2^ value for the HC group

Regions of interest	R^2^	Adjusted R^2^	Standard error of the estimate	*p*-value
MoCA at scan				
Right lateral temporal cortex, right mesial temporal cortex, right parietal cortex	0.405	0.337	1.120	0.003
MoCA 1 year after scan				
No model found				
MoCA 2 years after scan				
No model found				

Including the APOE status as an additional dependent variable in the linear regression did not improve the models. Using the ‘stepwise’ method of adding the APOE status as an additional dependent variable alongside the 20 ROIs resulted in the APOE variable being dropped from each model. When the APOE status was included as a variable using the ‘enter’ method alongside the other ROIs that were found using a stepwise model for each year, the adjusted R^2^ increased slightly for all the 3 years. However, the APOE variable was not significant (*p* > 0.05) as a coefficient in any of the models, meaning the models with APOE status were less precise despite any slight increase in adjusted R^2^.

## Discussion

We used idiopathic PD patients to elucidate the role Aβ accumulation has on PD cognitive decline using both ‘top-down’ and ‘bottom-up’ analysis methods. We found that Aβ accumulation has a moderate association with future cognitive decline in PD, but only when specific brain regions are the target of this increased Aβ burden. Conversely, Aβ burden in HC only moderately predicted present cognitive scores and had no prospective utility. We also found that Aβ deposition in PD differed from HC in its clustering. In PD, Aβ deposition formed 3 major cortical clusters, with Aβ deposition in the more posterior located cluster 2 being more strongly related with cognitive decline than the more anterior-ventral cluster 1. In HC, 2 of the clusters were made up of the bilateral ACC and bilateral PCC respectively, with the other 16 ROIs forming one large, more heterogenous cluster.

Cluster analysis is a tool that helps identify variables (i.e., brain regions) sharing similarity in some fashion [46, 47]. In PD cluster 1, the bilateral gyrus recti surprisingly had a significant positive Pearson correlation with MoCA score 1 year after scan, this means that in these frontal regions, cognitive performance was not negatively impacted by Aβ burden. The role of the rectus gyri in human cognition remains unclear, but it is hypothesized to be involved in emotional regulation, compulsive behaviour, and social cognition [48, 49, 50]. These cognitive abilities are not measured by the MoCA which could also explain why Aβ burden in this brain region was not associated with a cognitive decline. Alternatively, the positive relationship between Aβ in the left gyrus rectus and cognitive ability may suggest that this brain region may not significantly impact cognitive performance in the moderate stages of PD pathology.

We speculate that cluster 2 ROIs in the PD group are those most vulnerable to Aβ burden leading to cognitive decline in PD. The right parietal cortex and left occipital cortex had negative Pearson correlations with MoCA score at time of scan and 2 years after scan, respectively. Uribe et al. [44] performed a hierarchical cluster analysis on MRI derived cortical thickness data in 77 PD patients and found two patterns of cortical atrophy. PD patients grouped under the more posterior atrophy cluster had more atrophy in the bilateral occipital and superior parietal lobes as well as more pronounced cognitive decline as measured by a neurocognitive test battery, compared to PD patients with the more anterior atrophy cluster.

The bilateral PCC was unusual in our analysis, forming its own cluster in both PD and HC. The PCC region is a central node in the default mode network (DMN) and is densely connected with numerous other brain regions [51]. Previous studies have noted the association between Aβ deposition overlapping with nodes in the DMN [52, 53, 54]. In Alzheimer’s disease, it was found that Aβ deposition begins in the precuneus, medial orbitofrontal cortex, and PCC, which are all part of the DMN [53]. One hypothesis to this observation is that synaptic activity may drive Aβ deposition, and thus a lifetime of DMN activation would result in Aβ being deposited along the highly active DMN brain regions [55]. Sepulcre et al. [56] performed a longitudinal study examining how Aβ spread in HCs using another radiotracer that can measure cortical Aβ plaques in the brain, Pittsburgh compound B (PiB). Using graph theory analysis methods, they found that the PCC region acted as seed region for the spread of Aβ to neighbouring posterior and lateral parietal brain regions, namely the lateral fronto-parietal, midline frontal, and precuneus brain regions. In our study, these brain regions were all part of cluster 2 in PD. Greater Aβ burden in these regions may be working synergistically with PD-mediated dysfunctional α-synuclein and tau networks to create the conditions for PD cognitive decline [21].

The stepwise linear regression analysis found models which explain between 14.8 and 49.5% of the variance in PD MoCA score across different years. Interestingly, the strongest model with the highest adjusted R^2^ was found for MoCA score 1 year after the [^18^F]FBB scan in the PD group, suggesting in the potential prognostic strength in using this model to assess future cognitive decline in PD patients. These findings are in line with previous studies which found that measuring Aβ levels in the CSF were a good predictor of future cognitive decline in PD [22]. Gomperts et al. [11] found that high Aβ deposition in the precuneus was not able to distinguish PD-MCI from PD-CU at time of the scan. However, patients with higher baseline Aβ deposition and PD-MCI categorization experienced a more severe cognitive decline 2.5 years after scan compared to baseline PD-CU or low Aβ burden. These findings have since been replicated with numerous other longitudinal studies showing higher baseline CSF Aβ is predictive of future PD cognitive decline and dementia risk [57, 58, 59, 60, 61, 62, 63, 64, 65]. The weaker model found 2-years post-scan compared to 1-year post-scan is likely due to the additional covariates that arise from having more time to account for in the predictive model. In addition to beta-amyloid burden, widespread neurotransmitter dysregulation and grey matter atrophy are also likely involved in PD cognitive decline [8], all of which have a heterogenous progression in PD patients. In other words, it is easier to predict the near future than the far future because there are fewer variables to account for (i.e., the outcome 2-years post-scan relies on the outcome of 1-year post-scan, amplifying the unpredictability).

The right parietal cortex was found in the models predicting cognitive decline in PD both in the 1-year and 2-year follow-ups as well in the model predicting cognitive decline in HC at time of scan. The right parietal cortex is involved in attentional integration of sensory information for both halves of the body [66] and has been implicated in cognitive decline in PD using various imaging modalities. Structural MRI imaging studies have found increased cortical atrophy of the right parietal cortex in the brains of PD-MCI patients [67, 68] while functional FDG-PET found reduced metabolism in the bilateral parietal cortex correlated with reduced cognitive abilities [69, 70]. The correlation between the right parietal cortex in both the PD and HC groups’ with MoCA scores suggests it is a key brain region involved in Aβ mediated cognitive decline and may be particularly vulnerable to Aβ deposition.

In the HC group, the three clusters had notable differences from PD: while cluster three encompassed also the bilateral PCC, cluster two consisted of the bilateral ACC, and cluster one included the remaining 16 cortical ROIs together. Aβ deposition in neither the ACC nor the PCC had a significant correlation with MoCA score in our study. Instead, it was cluster one, specifically the right temporal cortices and right parietal cortex, which correlated with MoCA score at time of scan. This larger 16 ROI cluster found in HCs may suggest that an even distribution of Aβ deposition in the brain is likely healthier than localized deposits. Aβ plays a vital role in healthy brain function but is neurotoxic in both too high and too low amounts. If reaching a high threshold of Aβ in a brain region is pathological, a “subthreshold” distribution of the Aβ may not be so deleterious.

A linear regression model of the HC group was only found at time of scan with an R^2^ of 0.337 which explains just over a third of the variance in MoCA score. This model consisted of the right parietal cortex, right lateral temporal cortex and right mesial temporal cortex. The temporal cortices are involved in language processing, semantics, and memory encoding [71, 72] which are all cognitive aspects measured by MoCA. Failing to find a linear regression model 1-year and 2-year post-scan suggests Aβ deposition mediated cognitive decline may work differently in HC than in the presence of PD pathology. The lack of accompanying brain pathologies in HC in the form of α-synuclein and neuroinflammation dysregulation present in PD pathology may offer a protective effect in which high Aβ levels alone may be insufficient for triggering cognitive decline.

We included only idiopathic PD patients in our sample to minimize the confounding effects genetic variants would have on our data. We did not find APOE status as a relevant variable in our linear regression analysis, however this is likely due to the small sample size of each allele variant.

When examining Aβ pathology, it is common for studies to examine an Aβ metric that measures global or composite Aβ burden in the brain as a binary marker of having too much Aβ in the brain (Aβ+) or not (Aβ−) [29]. Many different studies have proposed different cut-off numbers of SUVR to classify Aβ+ [12, 13, 31, 40], which vary by radiotracer type, scanner resolution, disease pathology, method of comparing radiotracer uptake, and reference region [73, 74, 75]. However, we propose that having a high composite level of Aβ in the brain is not telling the complete picture regarding amyloid burden in the brain. We found that having a high level of Aβ in some parts of the brain, such as the bilateral rectus gyrus, may be less detrimental to cognitive function. These brain regions are often included in a cortical composite score quantifying Aβ positivity [76, 77, 78], thus contributing towards Aβ+ designation while also being less pathological. This may explain also why some studies have failed to find a difference in PD cognitive status between Aβ+ and Aβ− groups.

The present study has some limitations that ought to be addressed. First, we only used MoCA score to quantify cognitive ability instead of a more comprehensive neuropsychological test battery that would measure cognitive ability in different domains. We used the MoCA due to its popularity as a screening tool for PD-MCI [38] allowing for an easier time replicating these results in future studies. However, analyzing how specific cognitive domains are affected by cortical Aβ deposition may be an interesting follow-up. Second, there are also limitations due to the nature of the PPMI study protocol that we could not control for. The PET imaging data we used was collected from several different sites with different PET cameras which can affect the outcome measure of SUVR. We also had far more males than females in the PD group, as well as more PD-CU patients than PD-MCI especially in the follow-up years due to fact that the most pathological patients tend to drop out first, and this may have also impacted our results. Furthermore, although PPMI enrollment began with de novo PD patients, most patients were already on treatment by the time they were scanned with [^18^F]FBB radiotracer. Finally, we only used the Aβ values at baseline, but it would also be useful to measure Aβ in the yearly follow-ups parallel to the cognitive measurements to get a more comprehensive understanding of Aβ changes in concert with the cognitive decline.

## Conclusion

Our results suggest that local Aβ burden in PD alone can predict nearly half of the variance in MoCA score in the year after scan. Thus, Aβ burden is necessary, but not sufficient, towards explaining the cognitive decline symptoms in PD. Higher Aβ burden in cluster 2 ROIs was a stronger predictor of cognitive decline than higher Aβ burden in clusters 1 or 3. Furthermore, cortical Aβ burden had only a weak effect on cognition in the year Aβ density is measured but had a stronger effect on cognition in the years post-scan. We propose measuring the local impact of Aβ burden on specific ROIs rather than a whole-brain global Aβ composite score to measure the extant impact of Aβ on PD cognitive decline and use this information as a tool for determining which PD patients are most at risk for future cognitive decline.

## Supplementary Information

**Additional file 1: Table S1.** Genetic alleles with Parkinson disease-associated variants for GBA, SNCA and LRRK2 alleles that were excluded in the Parkinson’s disease sample; all included Parkinson’s disease patients did not have these pathogenic alleles.

**Additional file 2.** Additional results provided for the Durbin–Watson tests used in the linear regression model as well as the full linear regression equations for each year.

**Additional file 3: Figure S1.** The tables below show the Pearson correlation coefficient values of each [^18^F]Florbetaben ‘standardized uptake value ratios (SUVR)’ × ‘Montreal Cognitive Assessment scores (MoCA)’ at the different years for (A) Parkinson’s disease patients on top and (B) healthy controls below, with the (C) Spearman correlation additionally calculated for the non-normally distributed healthy control groups. Cells marked with an asterisk are statistically significant (*p* < 0.05) and are highlighted in green for a positive correlation or cyan for a negative correlation. The three clusters are colour coded in orange for cluster 1, purple for cluster 2, and red for cluster 3.

**Additional file 4: Table S2.** Statistical tests for the regions of interest found in the linear regression model of Parkinson’s disease group.

**Additional file 5: Table S3.** Statistical test results for each region of interest used in the stepwise linear regression model for the healthy control group.

## Data Availability

The datasets supporting the conclusions of this article are available online at the PPMI repository. Please visit https://www.ppmi-info.org/ to apply for access to this database which is open publicly for researchers to use.

## Abbreviations

PD: Parkinson’s disease
Aβ: Beta amyloid
PD-MCI: Parkinson’s disease with mild cognitive impairment
PDD: Parkinson’s disease dementia
PD-CU: Cognitively unimpaired Parkinson’s disease
CSF: Cerebrospinal fluid
Aβ+: Beta amyloid positive
Aβ−: Beta amyloid negative
ROI(s): Region(s) of interest
SUVR(s): Standard uptake value ratio(s)
PCC: Posterior cingulate cortex
ACC: Anterior cingulate cortex
PET: Positron emission tomography
[^18^F]FBB: Fluorine-18 florbetaben
PPMI: Parkinson’s Progression Marker Initiative
HC: Healthy control
MoCA: Montreal Cognitive Assessment
GBA: β-Glucocerebrosidase
SNCA: α-Synuclein
LRRK2: Leucine-rich repeat kinase 2
APOE: Apolipoprotein E
MDS‐UPDRS: Movement Disorders Society‐Unified Parkinson Disease Rating Scale
H&Y: Hoehn and Yahr
GDS: Geriatric Depression Scale
LEDD: Levodopa equivalent daily dose
MNI: Montreal Neurological Institute
SUV(s): Standard uptake value(s)
VOI(s): Volume(s) of interest
AAL: Automated Anatomical Labeling
DMN: Default mode network
PiB: Pittsburgh compound B
