# Supplementary material for: Beta amyloid deposition and cognitive decline in Parkinson’s disease: a study of the PPMI cohort
Source: Mol Brain. 2022 Sep 13;15:79. doi: 10.1186/s13041-022-00964-1 (PMC9472347; doi:10.1186/s13041-022-00964-1)
Supplement: Supplementary file 1 — Additional file 1: Table S1. Genetic alleles with Parkinson disease-associated variants for GBA, SNCA and LRRK2 alleles that were excluded in the Parkinson’s disease sample; all included Parkinson’s disease patients did not have these pathogenic alleles. [file 13041_2022_964_MOESM1_ESM.docx]

| **Reference SNP cluster ID (rsid)** | **Chromosome** | **BP hg38** | **Variant name /implicated gene(s)** |
| --- | --- | --- | --- |
| **GBA** | | | |
| rs421016 | 1 | 155235252 | GBA_L444P |
| rs76763715 | 1 | 155235843 | GBA_N370S |
| rs75548401 | 1 | 155236246 | GBA_T408M |
| rs2230288 | 1 | 155236376 | GBA_E365K |
| rs104886460 | 1 | 155240629 | GBA_IVS2+1 |
| rs387906315 | 1 | 155240660 | GBA_84GG |
| **SNCA** | | | |
| rs104893877 | 4 | 89828149 | SNCA_A53T |
| rs104893875 | 4 | 89828170 | SNCA_E46K |
| rs104893878 | 4 | 89835580 | SNCA_A30P |
| **LRRK2** | | | |
| rs76904798 | 12 | 40220632 | LRRK2 |
| rs33939927 | 12 | 40310434 | LRRK2_R1441G |
| rs33939927 | 12 | 40310434 | LRRK2_R1441C |
| rs33949390 | 12 | 40320043 | LRRK2_R1628P/H |
| rs35801418 | 12 | 40321114 | LRRK2_Y1699C |
| rs34637584 | 12 | 40340400 | LRRK2_G2019S |
| rs34778348 | 12 | 40363526 | LRRK2_G2385R |
|  |  |  |  |

**Table S1** Genetic alleles with Parkinson disease-associated variants for GBA, SNCA and LRRK2 alleles that were excluded in the Parkinson’s disease sample; all included Parkinson’s disease patients did not have these pathogenic alleles.
