# Supplementary material for: Beta amyloid deposition and cognitive decline in Parkinson’s disease: a study of the PPMI cohort
Source: Mol Brain. 2022 Sep 13;15:79. doi: 10.1186/s13041-022-00964-1 (PMC9472347; doi:10.1186/s13041-022-00964-1)
Supplement: Supplementary file 2 — Additional file 2. Additional results provided for the Durbin–Watson tests used in the linear regression model as well as the full linear regression equations for each year. [file 13041_2022_964_MOESM2_ESM.docx]

# Additional Results

In PD at time of scan, a stepwise linear regression found a model consisting of the left occipital cortex with an adjusted R^2^ of 0.148 and a Durbin-Watson of 2.267 (D_U_ for N = 25 with 2 terms = 1.454, D > D_U_ , [4 – D] > D_U_). PD MoCA score at time of scan was = 42.787 – 11.793 (left occipital cortex SUVR).

One year after scan, a stepwise linear regression found a model consisting of the left gyrus rectus, right parietal cortex and left anterior cingulate cortex with an adjusted R^2^ of 0.495 and a Durbin-Watson of 2.158 (D_U_ for N = 23 with 4 terms = 1.660, D > D_U_ , [4 – D] > D_U_). PD MoCA score one year after scan was = 28.568 + 25.167 (left gyrus rectus SUVR) - 14.698 (right parietal cortex SUVR) – 10.892 (left anterior cingulate cortex SUVR).

Two years after scan, a stepwise linear regression found a model consisting of the left gyrus rectus and right parietal cortex with an adjusted R^2^ of 0.319 and a Durbin-Watson of 1.883 (D_U_ for N = 21 with 3 terms = 1.538, D > D_U_ , [4 – D] > D_U_). PD MoCA score two years after scan was = 36.767 – 19.720 (right parietal cortex SUVR) + 12.996 (left gyrus rectus SUVR).

In HC at time of scan, a stepwise linear regression found a model consisting of the right lateral temporal cortex, right mesial temporal cortex, and right parietal cortex with an adjusted R^2^ of 0.337 and a Durbin-Watson of 1.708 (D_U_ for N = 30 with 4 terms = 1.650, D > D_U_ , [4 – D] > D_U_). ). HC MoCA score at time of scan = 35.532 – 21.943 (right lateral temporal cortex SUVR) + 7.616 (right mesial temporal cortex SUVR) + 8.325 (right parietal cortex SUVR).
