# Supplementary material for: Beta amyloid deposition and cognitive decline in Parkinson’s disease: a study of the PPMI cohort
Source: Mol Brain. 2022 Sep 13;15:79. doi: 10.1186/s13041-022-00964-1 (PMC9472347; doi:10.1186/s13041-022-00964-1)
Supplement: Supplementary file 3 — Additional file 3: Figure S1. The tables below show the Pearson correlation coefficient values of each [18F]Florbetaben ‘standardized uptake value ratios (SUVR)’ × ‘Montreal Cognitive Assessment scores (MoCA)’ at the different years for (A) Parkinson’s disease patients on top and (B) healthy controls below, with the (C) Spearman correlation additionally calculated for the non-normally distributed healthy control groups. Cells marked with an asterisk are statistically significant (p < 0.05) and are highlighted in green for a positive correlation or cyan for a negative correlation. The three clusters are colour coded in orange for cluster 1, purple for cluster 2, and red for cluster 3. [file 13041_2022_964_MOESM3_ESM.pdf]

A

	Cluster 1										Cluster 2								Cluster 3	
	L temporal cortex	L lateral temporal cortex	L mesial temporal cortex	R mesial temporal cortex	R temporal cortex	R lateral temporal cortex	L orbito-frontal cortex	R orbito-frontal cortex	L gyrus rectus	R gyrus rectus	L parietal cortex	R parietal cortex	L occipital cortex	R occipital cortex	L frontal cortex	R frontal cortex	L ACC	R ACC	L PCC	R PCC
MoCA at scan	-0.382	-0.381	-0.356	-0.234	-0.271	-0.271	-0.200	-0.135	-0.044	0.054	-0.345	-0.269	**-0.428***	-0.392	-0.324	-0.291	-0.364	-0.263	-0.126	-0.024
MoCA one year after scan	0.247	0.211	0.309	0.324	0.090	0.013	0.329	0.245	**0.424***	**0.419***	-0.293	-0.359	-0.151	-0.149	-0.109	-0.169	-0.170	-0.145	-0.255	-0.401
MoCA two years after scan	0.009	-0.014	0.079	0.129	-0.126	-0.200	0.169	0.100	0.310	0.345	-0.357	**-0.459***	-0.296	-0.326	-0.207	-0.258	-0.248	-0.163	-0.141	-0.208

B

	Cluster 1																Cluster 2		Cluster 3	
	L mesial temporal cortex	R mesial temporal cortex	L temporal cortex	R temporal cortex	L lateral temporal cortex	R lateral temporal cortex	L orbito-frontal cortex	R orbito-frontal cortex	L gyrus rectus	R gyrus rectus	L frontal cortex	R frontal cortex	L parietal cortex	R parietal cortex	L occipital cortex	R occipital cortex	L ACC	R ACC	L PCC	R PCC
MoCA at scan	-0.239	-0.217	-0.302	**-0.395***	-0.325	**-0.447***	-0.265	-0.324	-0.325	-0.249	-0.351	-0.352	-0.250	-0.281	-0.303	-0.316	-0.343	-0.285	-0.156	-0.147
MoCA one year after scan	-0.027	-0.002	-0.003	-0.068	-0.013	-0.094	-0.184	-0.134	-0.204	-0.195	-0.236	-0.251	-0.199	-0.254	-0.171	-0.179	-0.415	-0.304	-0.193	0.292
MoCA two years after scan	-0.182	-0.103	-0.093	-0.161	-0.072	-0.193	-0.157	-0.169	-0.118	-0.095	-0.206	-0.218	-0.196	-0.196	-0.275	-0.240	-0.267	-0.228	-0.199	-0.214

C

	Cluster 1																Cluster 2		Cluster 3	
	L mesial temporal cortex	R mesial temporal cortex	L temporal cortex	R temporal cortex	L lateral temporal cortex	R lateral temporal cortex	L orbito-frontal cortex	R orbito-frontal cortex	L gyrus rectus	R gyrus rectus	L frontal cortex	R frontal cortex	L parietal cortex	R parietal cortex	L occipital cortex	R occipital cortex	L ACC	R ACC	L PCC	R PCC
MoCA at scan	-0.110	-0.095	-0.156	-0.302	-0.144	**-0.378***	-0.155	-0.236	-0.265	-0.172	-0.230	-0.234	-0.143	-0.182	-0.209	-0.183	-0.276	-0.195	-0.032	-0.034
MoCA one year after scan	-0.067	-0.153	-0.114	-0.196	-0.135	-0.171	-0.172	-0.164	-0.211	-0.120	-0.170	-0.149	-0.208	-0.185	-0.151	-0.199	-0.114	-0.127	-0.117	-0.229
MoCA two years after scan	-0.374	-0.319	-0.221	-0.402	-0.225	-0.356	-0.227	-0.320	-0.188	-0.181	-0.327	-0.309	-0.347	-0.407	-0.422	-0.427	-0.270	-0.254	-0.393	-0.429
