# Supplementary material for: Beta amyloid deposition and cognitive decline in Parkinson’s disease: a study of the PPMI cohort
Source: Mol Brain. 2022 Sep 13;15:79. doi: 10.1186/s13041-022-00964-1 (PMC9472347; doi:10.1186/s13041-022-00964-1)
Supplement: Supplementary file 4 — Additional file 4: Table S2. Statistical tests for the regions of interest found in the linear regression model of Parkinson’s disease group. [file 13041_2022_964_MOESM4_ESM.docx]

**Table S2** Statistical tests for the regions of interest found in the linear regression models of Parkinson’s disease group.

| Regions of interest | Unstandardized β | Coefficients  standard error | Standardized  coefficients β | t-statistic | *p*-value |
| --- | --- | --- | --- | --- | --- |
| **MoCA at scan** | | | | | |
| Constant | 42.797 | 6.941 |  | 6.164 | < 0.001 |
| Left occipital cortex | -11.793 | 5.188 | -0.428 | -2.273 | 0.033 |
| **MoCA one year after scan** | | | | | |
| Constant | 28.568 | 8.039 |  | 3.554 | 0.002 |
| Left gyrus rectus | 25.187 | 5.808 | 0.787 | 4.337 | < 0.001 |
| Right parietal cortex | -14.698 | 5.611 | -0.421 | -2.619 | 0.017 |
| Left anterior cingulum | -10.892 | 4.346 | -0.463 | -2.506 | 0.021 |
| **MoCA two years after scan** | | | | | |
| Constant | 36.797 | 10.364 |  | 3.548 | 0.002 |
| Right parietal cortex | -19.720 | 7.031 | 0.525 | -2.805 | 0.012 |
| Right gyrus rectus | 12.996 | 5.711 | 0.425 | 2.276 | 0.035 |
