# Supplementary material for: Beta amyloid deposition and cognitive decline in Parkinson’s disease: a study of the PPMI cohort
Source: Mol Brain. 2022 Sep 13;15:79. doi: 10.1186/s13041-022-00964-1 (PMC9472347; doi:10.1186/s13041-022-00964-1)
Supplement: Supplementary file 5 — Additional file 5: Table S3. Statistical test results for each region of interest used in the stepwise linear regression model for the healthy control group. [file 13041_2022_964_MOESM5_ESM.docx]

| Regions of interest | Unstandardized β | Coefficients  standard error | Standardized  coefficients β | t-statistic | *p*-value |
| --- | --- | --- | --- | --- | --- |
| **MoCA at scan** | | | | | |
| Constant | 35.532 | 2.481 |  | 14.321 | < 0.001 |
| Right lateral temporal cortex | -21.943 | 5.811 | -1.705 | -3.776 | 0.001 |
| Right mesial temporal cortex | 7.616 | 3.095 | 0.741 | 2.461 | 0.021 |
| Right parietal cortex | 8.325 | 4.043 | 0.696 | 2.059 | 0.049 |

**Table S3** Statistical tests for the regions of interest found in the linear regression model of the healthy control group.
